# “Fiddling While Rome Burns”: The Role of Ecological States in the Association Between Greenhouse Gas Emissions and Subjective Well-Being

**DOI:** 10.3389/fsoc.2020.00011

**Published:** 2020-02-28

**Authors:** Paola Signoretta, Piet Bracke, Veerle Buffel

**Affiliations:** ^1^School of Social Science, Loughborough University, Loughborough, United Kingdom; ^2^Department of Sociology, Ghent University, Ghent, Belgium; ^3^Department of Sociology, University of Antwerp, Antwerp, Belgium

**Keywords:** mental well-being, ecological state, greenhouse gas emissions, EQLS, regional analysis, European Union

## Abstract

Since March 2019, students around the world have taken to the streets to express their anger at the lack of effective actions against the threat of climate change, essentially accusing governments, and adults in general of “fiddling while Rome burns.” This paper puts forward the hypothesis that the ecological state moderates the positive association found in the literature between greenhouse gas emissions and mental well-being, taken as evidence of fiddling on climate change issues. This hypothesis is examined in the context of the countries and regions of the European Union using a hierarchical three-level analysis on the third wave (2011–2012) of the European Quality of Life Survey for a sample of EU citizens. The ecological state is operationalized using a climate change performance index. NO_2_ emissions data at the regional level are used as a measure of GHG emissions for regions of the European Union. The findings seem to support the hypothesis that individuals across ecological states keep “fiddling” on climate change issues as a trade-off between environmental and economic considerations. However, the mental well-being of the well-off is being eroded in moderate ecological states compared to good ecological states, which is a call for government to stop “fiddling” and act on climate change issues.

## Introduction

Since March 2019, students around the world have taken to the streets to express their anger at the lack of effective actions against the threat of climate change (Glenza et al., 2019), essentially accusing governments and adults in general of “fiddling while Rome burns.” More demonstrations organized by (adults) Extinction Rebellion (Taylor and Gayle, 2019) a British climate group, have also taken place around the globe. The protests highlight the fear for the doomed future of the earth if effective action is not taken promptly in the next ten year. Psychologists have identified “ecoanxiety” i.e., the “chronic fear of environmental doom” (Albrecht, 2011) as a psychological disorder that present serious mental consequences if not addressed appropriately (Clayton et al., 2017). There is however a paradox between the anxiety felt by individuals about climate change and the positive association found in the literature between greenhouse gas (GHG) emissions, a major contributor to climate change, and well-being. This positive association is taken as evidence that individuals keep “fiddling” on climate change issues.

Studies on the impact of climate change on mental health and well-being (Fritze et al., 2008; Berry et al., 2010; Clayton et al., 2017) have explored the “direct impacts of climate change on mental health due to trauma caused by extreme weather events for instance; and the indirect impacts caused by a changing climate on the social, economic, and environmental determinants of mental health.” (Fritze et al., 2008, p. 1) This paper contributes to this literature on climate change and mental well-being from a different two-fold perspective. Firstly, it focuses on the association between GHG emissions—major contributors to climate change—and mental well-being (Andersson et al., 2014; Fanning and O'Neill, 2019). GHG emissions (such as CO_2_ and NO_2_) derive mainly from economic activities and consumption practices (Keuken et al., 2015; IEA, 2017) both of which are linked to well-being through pathways that touch upon individuals' identity, for instance, through the employment opportunities offered by polluting industries and social comparisons expressed through lifestyle choices. “Decoupling” between economic growth and its environmental impacts and “downshifted” lifestyles have been put forward in different areas of research as two ways to reduce GHG emissions (Gough, 2016; Büchs and Koch, 2017) and slow down climate change along the way. Exploring the association between GHG emissions and mental well-being might provide evidence on how to approach *decoupling* and *downshifting* given the potential impacts on human well-being (Büchs and Koch, 2019; Fanning and O'Neill, 2019).

Secondly, studies on the association between GHG emission activities and subjective well-being have looked at either the individual level or the aggregate level of the association. However, little is known of the association in a multilevel context that is how individuals' mental well-being responds to the economic and policy context in which GHG emissions take place. Recent research found that states' environmental regimes (Duit, 2016) moderate the association between air pollution and mental well-being (Signoretta et al., 2019). Building on this recent work, it is hypothesized that the association between GHG emissions and mental well-being might be moderated by states' actions in the area of climate change.

Given the novelty of this perspective in the academic literature, this paper puts forward some initial hypotheses and—while recognizing the existing limitations of this work—it explores the potential for future research.

## Literature

Studies on the link between greenhouse gas (GHG) emissions, carbon footprints and people's well-being found mixed evidence. On the one hand GHG emissions from different household consumption areas and subjective well-being were found to be weakly associated in Sweden (Andersson et al., 2014) while higher carbon footprints were associated with marginally lower levels of well-being in Australia (Ambrey and Daniels, 2017). On the other hand countries with declining per capita consumption, measured in terms of either gross domestic product (GDP) or carbon footprint, show significant reductions in average happiness, though countries with growing per capita consumption have no significant change in happiness (Fanning and O'Neill, 2019). Moreover, Andersson et al. (2014) investigated the nature of the association to see how individuals with low GHG emissions and high SWB vary from other respondents and found that individuals holding materialist values have lower subjective well-being, while producing higher GHG emissions. Within this wider body of research, the focus is on individuals' values, environmental attitudes and consumption practices. A less researched side is whether the association between GHG and subjective well-being is dependent on the structural setting in which it takes place i.e. on states' environmental actions.

Recent work found that states' environmental regimes—that is states' commitments in terms of environmental policy, governance, and structures—moderate the association between air pollution and mental well-being (Signoretta et al., 2019). In particular, it appears that the perception of major air pollution problems in everyday life lowers mental well-being of people living in *partial* and *established* ecological states (Duit, 2016)—states characterized by least developed administrative organization and below average levels of regulation, taxation, and research and development spending the first and a well-develop administrative structure, taxation policies, and average levels of research spending in environmental issues the latter. As suggested in this recent work (Signoretta et al., 2019), if there is an influence on people' attitudes toward economic and environmental considerations due to the states' engagement (or lack of) in the climate change arena, then the association between GHG emissions and mental well-being might vary in different ecological states.

Based on this recent work, this paper develops a theoretical framework within which the association between GHG emissions and mental well-being can be interpreted in relation to states' actions in the area of climate change. It can be speculated that individuals who live in ecological states committed to combatting climate change are at a more advanced stage toward the acceptance of changes in social practices needed to move toward degrowth (Büchs and Koch, 2017) compared to individuals living in countries that could be defined as “in transition” toward climate protection. In this context individuals living in countries that are more ecologically committed and at a more advanced stage in the transition toward sustainability in terms of social practices (Büchs and Koch, 2017) might be more willing to accept a cut in their standard of living to curb the effects of climate change (as proposed by “decoupling” and “downshifting” proponents) which will result in a lessened impact on their well-being. Individuals in these countries might feel more protected from the negative consequences of climate change and might be less affected in terms of well-being because of the environmental actions undertaken by their governments in the long run. Based on this argument, living in (poor, in transition, or more advanced) ecological states might influence the strength of the association between GHG emissions and mental well-being. In addition in this range of ecological states, individuals' well-being might be affected differently depending on their different positions along the socio-economic scale.

As explained through the post-materialism theory, more affluent people in richer societies are more concerned with post-materialist values such as environmental protection (Inglehart, 1997; Fairbrother, 2012; Sulemana, 2016).

More specifically, the paper examines firstly whether GHG emissions have significant positive impacts on mental well-being (Hypothesis 1) as largely reported in the literature, secondly whether this association is strengthened or weakened by “good” ecological states in the area of climate change protection (Hypothesis 2). Thirdly, it is assessed whether the association varies by different socio-economic groups and how i.e., whether the well-being of the advantaged (disadvantaged) varies by regional GHG emissions (Hypothesis 3). Finally, it will be assessed whether the well-being of advantaged (disadvantaged) varies by ecological states' climate change actions (Hypothesis 4). The last two hypotheses will assess whether the well-being of the advantaged (disadvantaged) is more affected by the environmental quality measured in terms of GHG emissions or the climate change policies and actions undertaken by governments.

Methodologically, the paper also argues that for policy purposes it is important to conduct a comparative cross-national study (Signoretta et al., 2019)—as conducted in comparative cross-country health research (Brennenstuhl et al., 2011)—that also takes into account subnational (regional) conditions that are more significant to people's everyday lives. Thus, this work examines differences and similarities across ecological states in the European Union, in terms of climate change performance. In particular, it investigates whether ecological states moderate the association between GHG emissions and mental well-being and how different socio-economic groups are affected by GHG emissions and governments' climate change policies and actions.

## Data and Methods

Together with the conceptual innovation, the strength of this study lies in the analytical approach used. Three levels of analysis are employed namely individual, regional, and country levels. The individual data are provided by the European Quality of Life Survey (EQLS), wave 3 (2011–2012), which has a random sample of adult population resident in 34 countries including the current 28 EU member countries (as at October 2018)^1^. The survey provides valuable information on the living conditions and well-being of Europeans. For the analyses, a subsample of 21 countries is used, depending on the availability of information at the regional level regarding physical environmental conditions (air pollution, see below). One limitation of the EQLS database is however the lack of information on environmental preferences and attitudes of respondents. In order to supplement the lack of data on individuals' environmental preferences a range of individual characteristics are taken into account as they provide an indication of individuals' support for climate policies (Fritz and Koch, 2019, p. 1). The theoretical basis for this substitution of data is provided by the postmaterialist theory (Inglehart, 1995, 1997). Though some scholars have questioned the general validity of the theory, it is recognized that advantaged individuals (wealthier, better educated, in sociocultural employment etc.) are more concerned about the environment (Fairbrother, 2012; Sulemana, 2016). For the regional level, the second level of the Nomenclature of Territorial Units for Statistics (NUTS 2) is used. There was a certain degree of discrepancy between the regional information included in the EQLS dataset and the other data sources used for the macro level data (Eurostat, the EU statistical service; and OECD statistics). Consequently, matching was partly undertaken manually. After recoding, a total of 212 regions (NUTS 2 as baseline and if NUTS2 was not available then NUTS1) were used for analysis. As a result, the final sample contains information for 26,978 respondents.

GHG emissions are measured at the regional level as climate change affects individuals and communities by changing the physical environment in which they live. Climate policies and actions instead are primarily carried out at the national level, while the implementation is performed at a more local level. As an example, see Smart Cities, the European Commission's approach to improve the management and efficiency of the urban environment (European Commission, 2016).

### Measures

#### Individual and Local Level

The outcome variable mental well-being is measured by the 5-item scale (WHO-5) developed by the World Health Organization. The WHO-5 reflects both hedonic and eudemonic dimensions (Deci et al., 2008). The five items assess positive mood, vitality and general interest over the past 2 weeks and is an effective tool for revealing the frequency of depressive symptoms in the general population (Layte, 2012). Each answer is scored from zero to five and summed to produce a score out of 25. The scores in the EQLS data set are available rebased between 0 and 10. The higher the score the better the mental well-being of the respondent.

In order to capture socio-economic inequalities and environmental attitudes at the individual level, educational level, employment status, income and ownership of a house are taken into account. Education is a categorical variable, consisting of primary or less, secondary and tertiary education. Employment status has three categories: employed, unemployed, and non-employed (unable to work due to sickness/disability, retired, homemaker and student). The income level of respondents was assessed by relative equivalent household income, using the Modified OECD equivalence scale. To account for the high number of item non-responses, relative equivalent income was coded into five categories, with one category representing respondents with missing income data. Ownership of a house or apartment is used as a rough proxy for wealth, as no other information was available. The ownership of a house variable distinguishes between those who own a house, those who own a house with a loan and those who rent a house (including the category “others”).

Individual control variables known to be associated with depression are included: gender as a dummy variable and age as a metric variable. Household type is assessed through a five-category variable (single, couple without children, single parent, couple with children, and other). Spatial control variables include the degree of urbanization which is measured using four categories (rural, village/small town, medium/large town, and city/suburb). Migration status is a categorical variable consisting of non-migrant, migrant from an EU country and migrant from a non-EU country.

#### Regional Level

For the regional physical environmental characteristics, NO_2_ information (2011–2012) available from the Organization for Economic Co-operation and Development (OECD, 2019) is used. NO_2_ is selected as an indicator of human contribution to climate change and as a proxy of economic activities in a region. In addition, the use of this indicator instead of CO_2_ emissions avoids overlaps with the Climate Change Performance Index classification which includes CO_2_ emissions in the construction of the index (See below).

To test whether NO_2_ emissions are a proxy for regional macroeconomic conditions, Gross domestic product (GDP), [expressed in purchasing power standards (PPS)], Real growth rate of regional gross value added (GVA) at basic prices (percentage change on previous year) and the unemployment rate are used. Information was retrieved from Eurostat, the statistical office of the European Commission.

#### Country Level

**Figure 1:**
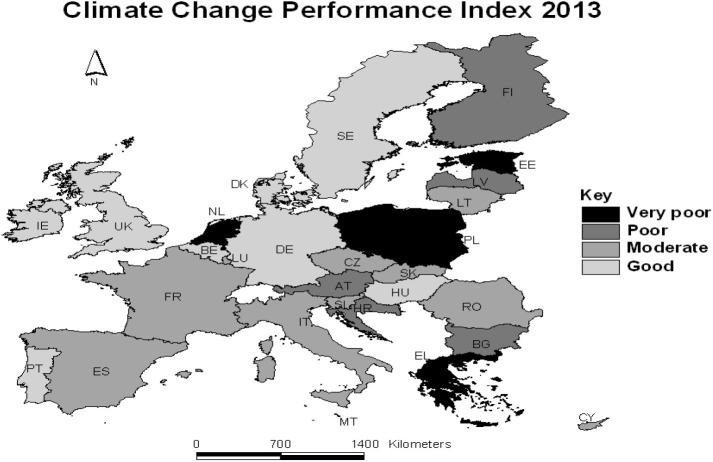
Classification of the 28 EU countries, as quantified by the Climate Change Performance Index (CCPI) for the year 2013 [CCPI: German Watch, (Burck et al., 2012); Administrative boundaries: ^©^EuroGeographics].

To measure the ecological performance of a country, the Climate Change Performance Index (CCPI) for the year 2013 was used. CCPI is available for 58 countries responsible for 90% of global energy-related CO_2_ emissions (Burck et al., 2012). It is measured via 15 different indicators that are combined into one single composite indicator. The indicators are classified into four categories (weighting in brackets): (1) Emissions: Emissions Level (30% weighting) Emissions Development (30% weighting); (2) Efficiency (10% weighting); (3) Renewable Energy (10% weighting); (4) Policy (20% weighting) (Burck et al., 2016). The CCPI ranking is used in relative rather than absolute terms (Burck et al., 2016). A categorical variable with three categories was constructed, namely: good, moderate, and poor (which includes poor and very poor). Figure 1 shows countries in each category. To take into account macro-economic conditions, GDP per capita is included at the country level.

### Analytical Strategy

The method section consists of a multilevel analysis performed on the total sample, using a hierarchical three level framework: individuals are nested in regions which are nested in countries. Multilevel analysis enables researchers to take the clustering of the data in regions and countries into account, and it allows us to estimate the impact of the GHG emissions on mental well-being.

Seven models are estimated to test our hypotheses, though depending on the significance of the results a selection of the models are reported in the results section.

The first model is a basic model, including the individual control and socio-economic variables only. This is used as a reference model to assess improvements of subsequent models. In the second model, regional NO_2_ is added to test the hypothesis that the association between regional GHG emissions and mental well-being is positive as expected in most literature. In the third model the regional and country macroeconomic variables are included to see whether the relation between regional NO_2_ emissions and mental well-being can be ascribed to regions' and countries' macroeconomic conditions. This model will ascertain whether the NO_2_ variable is a proxy for economic conditions of a region. The fourth model consists of the categorical variable CCPI. This model tests whether countries' ecological performance has an effect on mental well-being in particular whether subjective well-being is better in good ecological states. In the fifth model, a cross level interaction is tested between the NO_2_ emissions indicator and CCPI. This model tests whether the association between NO_2_ emissions and mental well-being varies by countries' ecological performance. Finally, two cross level interaction models are estimate between socio-economic position and NO_2_ and CCPI (sixth models and seventh model, respectively). The first model tests whether the association varies by different socio-economic groups while the second model tests how the well-being of individuals along the socio economic scale is affected by climate change ecological states. All models were estimated in MLwiN (Charlton et al., 2017).

## Results

### Descriptive Results

Table 1 presents the mental well-being mean scores by the categorical individual socio-economic and control variables. Appendix shows the relevant correlation coefficients between the mental well-being variable and regional (NO_2_, unemployment rate and GDP) and national (GDP per capita) variables. All coefficients are significant but very weak, except for the coefficients of the macroeconomic variables which are moderate and significant.

**Table 1:** Mental well-being by individual variables.

	**Mental well-being**	
	**Mean**	**SD**
**GENDER**		
Women	6.127	2.059
Men	6.460	1.975
**MIGRATION STATUS**		
Native	6.271	2.029
Migrant from EU	6.213	2.046
Non eu migrant	6.358	2.052
**HOUSEHOLD TYPE**		
Single	6.088	2.139
Couple without children	6.480	1.984
Single parent	5.652	2.133
Couple with children	6.381	1.846
Other	6.171	2.083
**TYPE OF COMMUNITY**		
The open countryside	6.494	1.994
A village/small town	6.195	2.034
A medium to large town	6.305	2.036
A city or city suburb	6.263	2.023
**EDUCATION**		
Primary or less	5.819	2.297
Secondary	6.258	2.037
Tertiary	6.537	1.809
**EMPLOYMENT STATUS**		
Employed	6.432	1.861
Unemployed	5.851	2.177
Non-employed	6.171	2.153
**INCOME**		
Below 50% median	5.696	2.325
Between 50 and 80% median	5.881	2.160
Between 80 and 120% median	6.256	1.966
Above 120% median	6.608	1.824
Missing	6.370	2.029
**HOUSE OWNERSHIP**		
Own a house	6.257	2.055
Own with loan	6.450	1.908
Rent and other	6.152	2.071

**Figure 2:**
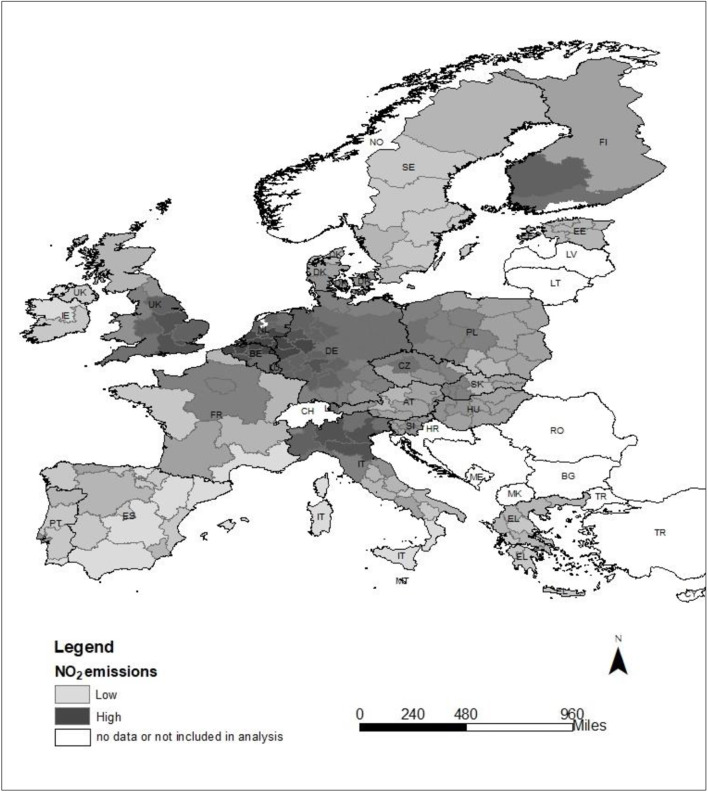
2011 NO_2_ (10^n^ mol/cm^2^) emissions across EU countries by NUTS2 regions (Source: OECD).

To start with, individuals who live in the countryside, have an income above 120% median and have tertiary education have higher mental well-being (Table 1). Moreover, there is a positive relation between NO_2_ emissions and mental well-being (Appendix) which might be explained by the geographical distribution of NO_2_ emissions in EU countries. As shown in Figure 2, the distribution of high and low areas of NO_2_ emissions across EU regions is quite clear-cut. Regions of high NO_2_ emissions are found in Belgium, Germany, Netherlands, UK and Northern Italy, regions performing well economically, which might explain the positive association between NO_2_ emissions and mental well-being, noted above.

As far as macroeconomic conditions are concerned, higher GDP per capita at the country level and regional GDP are associated with better mental well-being. As expected, higher GDP per capita at national level and regional GDP are associated with worse GHG emissions at regional as measured by NO_2_ emissions. Higher unemployment levels (at country and regional level) are associated with lower NO_2_ emission, and lower national and regional GDP and lower levels of mental well-being. Not surprisingly, better macroeconomic conditions (higher GDP levels and lower unemployment rates) are related to higher GHG emission levels, which is explained in the light of the current economic model characterized by the link between economic development and environmental damage (Büchs and Koch, 2017).

In terms of ecological performance (Figure 1), Belgium, Germany, Denmark, Hungary, Ireland, Portugal, Sweden and the UK are classified as “good” ecological state using the CCPI. Czech Republic, Spain, France, Italy, Luxembourg, Slovenia and Slovakia perform ecologically at a “moderate” level. Austria, Greece, Finland, The Netherlands and Poland are classified as “poor” ecological states. The very poor performance of the Netherlands stands out among the other neighboring countries and is explained in relation to its inadequate climate policy (Burck et al., 2012), while Lithuania moderate performance stands out among (the poor performance) of the surrounding Eastern European countries. Table 2 shows countries' climate change performance cross-classified against macroeconomic conditions and the GHG variable. Countries classified as “good,” however, present higher levels of NO_2_ emissions as these are also countries of high GDP. This may indicate a discrepancy between the environmental performance in terms of NO_2_ emissions and governance in countries classified as “good” on the CCPI. In contrast, in this group of countries, individuals have better mental well-being and the macroeconomic conditions are better. Countries' climate change performance is positively related to a lower average unemployment rate at the regional level, and a higher GDP per capita at country level and regional GDP.

**Table 2:** Mean scores on mental well-being, GHG emissions and macroeconomic indicators by countries' CCPI.

**Countries' CCPI**	**Good**		**Moderate**		**Poor**	
	**Mean**	**(SD)**	**Mean**	**(SD)**	**Mean**	**(SD)**
Mental well-being	6.42	(2.06)	6.20	(1.91)	6.13	(2.07)
Regional NO_2_	4.80	(2.77)	3.54	(1.73)	4.05	(2.19)
Regional GDP	2.92	(1.14)	2.20	(0.95)	2.24	(1.27)
Regional unemployment rate	8.67	(3.51)	11.17	(5.97)	9.11	(4.64)
National GDP per capita	27.91	(4.91)	23.84	(3.04)	23.65	(7.08)

### Multilevel Results

In the starting model, the individual socio-economic variables and control variables are included (model not shown). The higher educated, the employed, those with a higher income, men, and those who own a house have higher levels of mental well-being. In *model 1* (Table 3), the NO_2_ emissions variable at the regional level is included. In regions with a higher NO_2_ level, residents have better mental well-being. This confirms the hypothesis of a positive association between GHG emissions and mental well-being, as found in the literature.

**Table 3:** Mental Well-being regressed on NO_2_ emissions and macroeconomic variables.

	**Model 1**			**Model 2**		
	**b**	**S.E**.	***p***	**b**	**S.E**.	***p***
**FIXED PART**						
Intercept	6.279	0.106	^***^	6.338	0.100	^***^
**Income Group (Ref. Below 50%)**						
Between 50 and 80% median	0.144	0.052	^**^	0.138	0.057	0.015
Between 80 and 120% median	0.388	0.051	^***^	0.348	0.056	^***^
Above 120% median	0.640	0.052	^***^	0.572	0.057	^***^
**Regional level**						
NO_2_	0.050	0.016	^**^	0.031	0.017	
Regional GDP (× 10,000)				0.084	0.040	^*^
Regional unemployment 2011				0.004	0.010	
Regional GVA 2011				−0.007	0.011	
**National level**						
National GDP per capita (× 1,000)				0.010	0.008	
**RANDOM PART**						
Country	0.123	0.044		0.059	0.026	
Region	0.088	0.013		0.082	0.014	
Individual	3.763	0.033		3.774	0.036	
Country	21			17		
Region	218			180		
Individual	26,978			22,102		
**Estimation**						
−2LL	112,619			92315.4		

*All models take into account individual educational level, employment status, income and ownership of a house and control for age, gender, migration status and degree of urbanization of respondent residency*.

* *p < 0.05 level*,

** *p < 0.01 level*,

*** *p < 0.001*.

In *model 2* (Table 3), the macroeconomic conditions at regional level and country level are added. The NO_2_ indicator becomes not significant, which indicates that the association between emissions and mental well-being can be partly attributed to these macroeconomic factors. Also, the regional GDP variable is significant and positive.

Subsequently in *model 3* (Table 4), countries' ecological performance is included, but it has no significant main effect on mental well-being. In the next model, cross level interaction effects are estimated between country CCPI categories and NO_2_ emissions (model not shown), to test whether the relation between emissions and mental well-being varies according to countries' climate change ecological performance. However, the cross-level interaction terms between regional NO_2_ emissions and CCPI are not significant (model not shown). In *model 4*, the interaction terms between CCPI and socio-economic position (measured by income categories) are significant and negative for “moderate” CCPI countries compared to “good” CCPI counties. In sum, while income has a strong positive effect on mental well-being, the income effect is weaker in “moderate” CCPI countries compared to “good” CCPI countries. Moreover, the effect is more negative for higher income groups.

**Table 4:** Mental Wellbeing regressed on NO_2_, countries' CCPI and interaction terms.

	**Model 3**			**Model 4**		
	**b**	**S.E**.	***p***	**b**	**S.E**.	***p***
**FIXED PART**						
Intercept	6.451	0.142	^***^	6.374	0.148	^***^
**Income group (Ref. below 50%)**						
Between 50 and 80% median	0.144	0.052	^**^	0.261	0.078	^***^
Between 80 and 120% median	0.388	0.051	^***^	0.487	0.076	^***^
Above 120% median	0.640	0.052	^***^	0.724	0.075	^***^
**Regional level**						
NO_2_	0.048	0.016	^**^	0.049	0.016	^**^
**Country level**						
CCPI (ref. good) CCPI moderate	−0.298	0.184		−0.056	0.207	
CCPI Poor	−0.258	0.190		−0.240	0.216	
**INTERACTION TERMS**						
CCPI Moderate × between 50 and 80% median				−0.324	0.121	^**^
CCPI Poor × between 50 and 80% median				−0.066	0.130	
CCPI Moderate × between 80 and 120% median				−0.281	0.117	^*^
CCPI Poor × between 80 and 120% median				−0.049	0.125	
CCPI Moderate × above 120% median				−0.249	0.115	^*^
CCPI Poor × above 120% median				−0.039	0.124	
**RANDOM PART**						
Country	0.106	0.038		0.105	0.038	
Region	0.088	0.013		0.088	0.013	
Individual	3.763	0.033		3.761	0.033	
**OBSERVATIONS**						
Country	21			21		
Region	218			218		
Individual	26,978			26,978		
**Estimation:**						
−2LL	112616.31		112606.086			

*All models take into account individual educational level, employment status, income and ownership of a house and control for age, gender, migration status, and degree of urbanization of respondent residency*.

* *p < 0.05 level*,

** *p < 0.01 level*,

*** *p < 0.001*.

Finally, the interaction terms between NO_2_ emissions and socio-economic positions, estimated to test whether the relation between NO_2_ emissions and mental well-being varies by income groups, are not significant (model not shown).

## Discussion

This paper presents an exploratory examination of the complex relationship between regional GHG emissions and mental well-being in the context of climate change actions undertaken by countries within the European Union. To the best of our knowledge, there are no prior studies on the effects of countries' climate change actions in this association.

The theoretical framework assumes that people who live in ecological states committed to combatting climate change are at a more advanced stage toward the acceptance of changes in social practices^2^ needed to move toward degrowth (Büchs and Koch, 2017) compared to individuals living in countries that adopted moderate actions (thus “in transition”) toward climate protection. Following this reasoning, people living in more ecologically committed countries are at a more advanced stage in the transition toward climate change protection not only in terms of environmental actions but also in terms of lifestyle changes. Consequently, they might be more willing to accept a cut in their standard of living to curb the effects of climate change—as proposed by “downshifting” proponents—which will result in a lessened impact on their well-being. Individuals in these countries might feel more protected from the negative consequences of climate change and might be less affected in terms of well-being because of the environmental actions undertaken by their governments in the long run. Based on this reasoning, the argument put forward is that living in (poor, moderate or more advanced) ecological states might influence: (i) the strength of the association between GHG emissions and mental well-being and (ii) the well-being of individuals at along different socio-economic groups.

The results confirmed a positive association between GHG emissions as measured by regional NO_2_ levels and mental well-being (Hypothesis 1). It was also confirmed that this association can be attributed to macroeconomic factors. This means that overall in the EU countries included in this analysis, the NO_2_ emission variable can be considered a proxy for levels of economic activities and consumption practices in a region. This finding seems to confirm a trade-off between environmental and socio-economic considerations in the effect of NO_2_ emissions on mental well-being, as there is no evidence that individuals enjoy living in contaminated environments. While the analysis has not included variables associated with specific social practices which might reflect individuals' concerns about the environment, it has nevertheless controlled for socio-economic characteristics as individuals who are highly educated or employed in socio-cultural professions show support for climate change actions (Fritz and Koch, 2019).

However, the hypothesis that this positive association would be weaker in “good” climate change ecological states—as people awareness of negative effects of GHG emission would weaken the association—was rejected, as the interaction terms were not significant (Hypothesis 2). This is not surprising given that countries characterized by better economic performance and worst GHG emissions are also the most engaged in actions to combat climate change. Consequently, there is a discrepancy between climate change actions and environmental quality in terms of GHG emissions. This finding also provides more evidence of a trade-off between social and economic considerations.

Moreover, it was found that while living in countries differently committed to combatting climate change has no main effect on mental well-being, the mental well-being of higher socio-economic groups (as measured by income levels) in moderate CCPI countries is worse compared to good CCPI countries (Hypothesis 4). This finding indicates that the most economically advantaged individuals who live in “transition” countries in terms of climate change actions (i.e., those classified as moderate CCPI are expected to implement more advanced actions in the area of climate change, hence in transition) are the most affected. According to the post-materialist hypothesis (Inglehart, 1995, 1997) residents of richer countries are more willing to curb their standard of living to prevent environmental pollution and wealthier individuals care more about the environment compared to less advantage individuals. It can thus be speculated that while the adjustment to more ecologically sustainable social practices during the transition period might affect well-being negatively, in the instance of more socially advantaged (for instance in education and employment terms) individuals not addressing climate change issues might prove as negative for their well-being.

### Limitations

This study is based on a cross-sectional sample of European citizens drawn from the EQLS wave 3 for 2011 and a range of socio-economic and GHG data for the same period. As explained in the Data and Methods section, the wave 4 of the EQLS became available after the completion of the study. Given the exploratory nature of this work, the use of the third wave it is deemed not to compromise the theoretical framework on which this work is based. However, future work could explore the association for more recent time periods including the EQLS wave 4 for 2016 or employ longitudinal datasets.

While this work considered individual level factors which might influence one's own assessment of environmental conditions, including education level and place of residence, future research could be integrated with detailed information on people's environmental attitudes. Information on individual environmental attitudes are not included in the EQLS and consequently could not be employed in the analysis.

In addition, while the paper explores the effect of GHG emissions and CCPI on the well-being of different income groups, future work could utilize a range of indicators of socio-economic positions (education, employment etc.). Moreover, this work is also based on one objective measure of GHG emissions viz. NO_2_ emissions. Future work could use different measures of GHG emissions to assess whether the results of this preliminary work are confirmed.

In terms of the geographical unit of analysis, NUTS2 regions are quite large areas consequently individuals may not be directly affected or aware of the regional environmental conditions. However, there are several bottlenecks to the routine use of more detailed data in research that focuses on a cross-country and cross-region EU setting (Signoretta et al., 2019).

Finally, the paper focuses on climate change and uses a relevant climate change classification i.e., CCPI to identify different types of ecological states in relation to climate change actions. Future research could employ other relevant environmental state classifications in the climate change arena and/or the CCPI for different years. To address these limitations, it is recommended that further research on the association between GHG emissions, well-being and the role of the state is undertaken with the aim to explore these findings further in European countries and more diverse political, environmental, social and economic contexts.

## Conclusion

While the climate change demonstrations around the world show a level of unprecedented awareness and calls for governments to stop fiddling and act on climate change issues, the latest events demonstrate that these are polarizing issues (Taylor, 2019) with no straightforward ways to address them. As well-being is now recognized as one of the main objectives for policy, practice and research (Schwanen and Atkinson, 2015) assessing the well-being impacts that the actions undertaken by governments in terms of climate change (or for that matter any other environmental issue) have on individuals should become paramount. While economic growth and well-being are still seen as intertwined, the efforts to disjoin them must go hand in hand with a clear understanding of the effects on individuals' well-being. In this context, the path toward the development of a greener state (Eckersley, 2018) that will involve approaches such as degrowth (Fairbrother, 2012; Büchs and Koch, 2019) and decoupling (Coscieme et al., 2019) will have to proceed in ways that sustain levels of well-being in the transition period toward new greener social practices (Büchs and Koch, 2017). More research is indeed needed on the effects of these governments' environmental actions on the well-being of individuals.

## Data Availability Statement

Publicly available datasets were analyzed in this study. This data can be found here: https://www.eurofound.europa.eu/surveys/european-quality-of-life-surveys.

## Author Contributions

PS: conception and design of the work, theoretical framework, statistical analysis, interpretation of results, acquisition, analysis, and interpretation of results, drafting the work and revising it critically, final approval of the version to be published. PB: interpretation of results, revised manuscript critically, contribution to the design of work, final approval of the version to be published. VB: contribution to acquisition, analysis, and interpretation of data for the work, contributed to the statistical analysis, contributed to sections of the manuscript, and revised the work critically.

### Conflict of Interest

The authors declare that the research was conducted in the absence of any commercial or financial relationships that could be construed as a potential conflict of interest.

## Footnotes

^1^Wave 4 (2016) of the EQLS was not available at the time the study was conducted, hence wave three was used.

^2^For an examination of social practices, well-being and post-growth see Chapter 6 in Büchs and Koch (2017).

## Appendix

**Table:** Pearson correlation coefficients.

	**NO_2_**	**R_GDP**	**N_GDP**	**R_unemp**	**MWB**
NO_2_	–	0.286^**^	0.237^**^	–0.504^**^	0.048^**^
R_GDP	0.286^**^	–	0.330^**^	–0.368^**^	0.104^**^
N_GDP	0.237^**^	0.330^**^	–	–0.238^**^	0.076^**^
R_unemp	–0.504^**^	–0.368^**^	–0.238^**^	–	–0.044^**^
MWB	0.048^**^	0.104^**^	0.076^**^	–0.044^**^	–
**Key**	**Details**				
NO_2_	= NO_2_ emissions				
R_GDP	= Regional GDP				
N_GDP	= National GDP per capita				
R_unemp	= Regional unemployment rate				
MWB	= Mental Well-being				
